# Effect of an artificial intelligence-assisted community-based intervention on child restraint system practices in Shanghai, China

**DOI:** 10.3389/fpubh.2026.1799063

**Published:** 2026-04-20

**Authors:** Ning Gao, Xihui Wang, Shuna Gao, Juanjuan Peng, Yang Zheng, Bo Chen, Fengqian Qiu, Yan Yu, Weihua Chen

**Affiliations:** 1 Division of Noncommunicable Disease and Injury, Shanghai Municipal Center for Disease Control and Prevention, Shanghai, China; 2 Department for Prevention of Tumor and Injury, Shanghai Huangpu District Center for Disease Control and Prevention (Shanghai Huangpu District Health Supervision Institute), Shanghai, China

**Keywords:** artificial intelligence-assisted, child restraint system, community-based intervention, ownership rate, use rate

## Abstract

**Background:**

Child restraint systems (CRS) can effectively prevent injuries to children in road traffic crashes. However, in China, the rates of CRS ownership and use remain relatively low.

**Methods:**

We conducted a community-based intervention trial with an integrated multimodal intervention from August 2024 to March 2025 in five communities in Huangpu District, Shanghai. A total of 501 participants were recruited (201 in the intervention group, 300 in the control group). The intervention included (1) distribution of CRS educational brochures and health education materials, (2) dissemination of online educational articles, and (3) Artificial intelligence-assisted (AI-assisted) voice calls delivering standardized reminders and safety education. The control group received only pamphlets on dietary nutrition.

**Results:**

A total of 501 participants completed the study. After the intervention, the rate of CRS ownership showed no significant difference between the intervention and control groups (90.05% versus 91.33%, *p =* 0.74). However, the rate of consistent CRS use was significantly higher in the intervention group (50.75%) than in the control group (38.33%) (*p* < 0.01). The intervention group also showed fewer orientation-related installation errors than the control group (0.00% versus 12.55%, *p* < 0.01). Logistic regression with interaction terms indicated no significant intervention effect on ownership rate (OR = 0.97, 95% CI: 0.41–2.30), but a significant increase in consistent use rate (OR = 2.18, 95% CI: 1.25–3.82). Further analysis showed that ownership rate was associated with child age, parental education level, vehicle price, and travel frequency, while consistent use rate was associated with household registration, travel frequency, and travel distance.

**Conclusion:**

An integrated community-based intervention combining conventional health education with AI-assisted follow-up improved consistent CRS use and reduced orientation-related installation errors. This approach may be a useful complement to current CRS promotion strategies.

## 1 Introduction

Road traffic injury is a leading cause of mortality and disability among children globally. Each year, 186,300 children aged under 18 years of age lose their lives in road traffic crashes worldwide (1). In China, road traffic injury ranks as the second leading cause of death from injuries among children (2). As a critical strategy to mitigate this risk, child restraint systems (CRS) have proven highly effective. Evidence suggests that correct CRS use can reduce the risk of death by at least 60% among child passengers (3), and reduce the risk of fatal road traffic injuries among young children by approximately 54–80% (1). According to the World Health Organization (WHO), adopting legislation in line with best practice criteria is an important measure to increase CRS use (3, 4). International evidence confirms that mandatory CRS laws not only improve utilization rates but also markedly reduce traffic injury related hospitalizations and deaths (5, 6). However, China currently lacks national-level legislation with specific age, height, and weight requirements, and most regional regulations are primarily advisory. In Shanghai, the Regulations of Shanghai Municipality on Road Traffic Management (2017) state that CRS must be correctly used when transporting children under the age of four in family vehicles (7).

In China, the rates of CRS use vary significantly across different regions, ranging from 17.3–77.8% (8–10). In Shanghai, although the CRS ownership rate is relatively high at 86.77%, the use rate is merely 32.88%, indicating a prevalent gap between possession and consistent use (11). Similarly, in Shandong Province, while 56.1% of parents use CRS, only 29.0% reported consistent use (12), indicating that despite possession, many families have not established consistent CRS use habits.

CRS ownership and use are influenced by factors such as parental education, family income and travel distance (13). Misconceptions also play a crucial role. Research conducted in western China shows that a large proportion of parents (85.2%) mistakenly believe adult seat belts are sufficient for child safety, whereas the primary reported reason for non-use is children’s infrequent car travel (13). Misuse of CRS, particularly the failure to use the optimal restraint for a child’s developmental stage, is a common problem (14). These challenges - low possession, inconsistent use, and high misuse rates highlight the need for targeted interventions to change caregivers’ knowledge and attitudes towards CRS.

Legislation is important for increasing CRS use, but legislation alone may be insufficient to ensure correct use. More detailed legal requirements on the CRS type according to a child’s age, height, and weight are needed to help caregivers make appropriate choices (4). Educational and behavioral interventions are therefore also important. Previous studies have shown that some intervention approaches, including community-based, hospital-based and app-based models can improve parental knowledge, attitudes, and practices regarding CRS use (14–18).

With changes in the ways parents access health information, intervention approaches also need to change accordingly. It is important to examine whether integrating an AI-assisted, multimodal intervention into routine community health services can help address the persistent problems of CRS non-use and misuse in China. Therefore, this study aimed to evaluate the effect of an integrated intervention on CRS ownership, consistent use, and misuse. It also aimed to identify factors associated with CRS adoption and use among caregivers of young children in Shanghai, China.

## 2 Methods

### 2.1 Study design

We conducted a community-based intervention study with an integrated multimodal intervention from August 2024 to March 2025 in Huangpu District, Shanghai, China. Five communities were selected for participation. Of these, one community was selected as the intervention group, while the remaining four communities were selected as the control group. This allocation strategy was adopted based on population characteristics: the community selected for the intervention group possessed a larger service population, which facilitated the efficient, centralized implementation of intervention. The four control communities had relatively smaller populations and were geographically dispersed, making them suitable for comparison while minimizing potential interference between groups. Although the intervention and control communities were selected based on population size and geographic distribution. The non-random allocation may have introduced selection bias, baseline imbalance and residual confounding, which could affect the internal validity of the effect estimates. Doctors at the community health service center conducted questionnaire surveys and interventions for the parents of children who came for vaccination. The study employed a single-blind design, in which participants were unaware of their group allocation.

The protocol was approved by the Ethics Committee of Shanghai Huangpu District Center for Disease Control and Prevention (Shanghai Huangpu District Health Supervision Institute) (Approval No. HLQ202123).

### 2.2 Recruitment and participants

Participants were recruited from caregivers who brought their children to community health service centers for vaccination between August and September 2024. Those who agree to participate upon arrival were enrolled in the study and completed the baseline questionnaires. To be eligible for the study, participants were required to meet the following inclusion criteria: (a) being a parent of a child aged 0–3; (b) owning a private vehicle; (c) being able to read, write, and speak Chinese. Participants were excluded if they: (a) refused to participate in this survey; (b) did not use family cars as a mode of transport for their children; or (c) suffered from severe mental illness or cognitive impairment.

### 2.3 Data collection

Data were collected through face-to-face questionnaire surveys administered at baseline (August–September 2024) and post-intervention (March 2025). Participants signed informed consent forms both for the questionnaire and the intervention study. Participants in both the intervention and control groups completed the baseline survey prior to the implementation of any intervention measures.

The questionnaire collected data: (a) Demographic information: including gender, age, household registration, parents’ educational level; (b) Family vehicle usage: including frequency of private vehicle use, travel distance, vehicle price, and household income; (c) CRS outcomes: ownership and usage.

Child age was categorized into one-year intervals. Demographic and travel-related variables were grouped with reference to a previous Chinese study on CRS use (19), and then adapted to the questionnaire design of the this study.

### 2.4 Intervention

**Figure 1:**
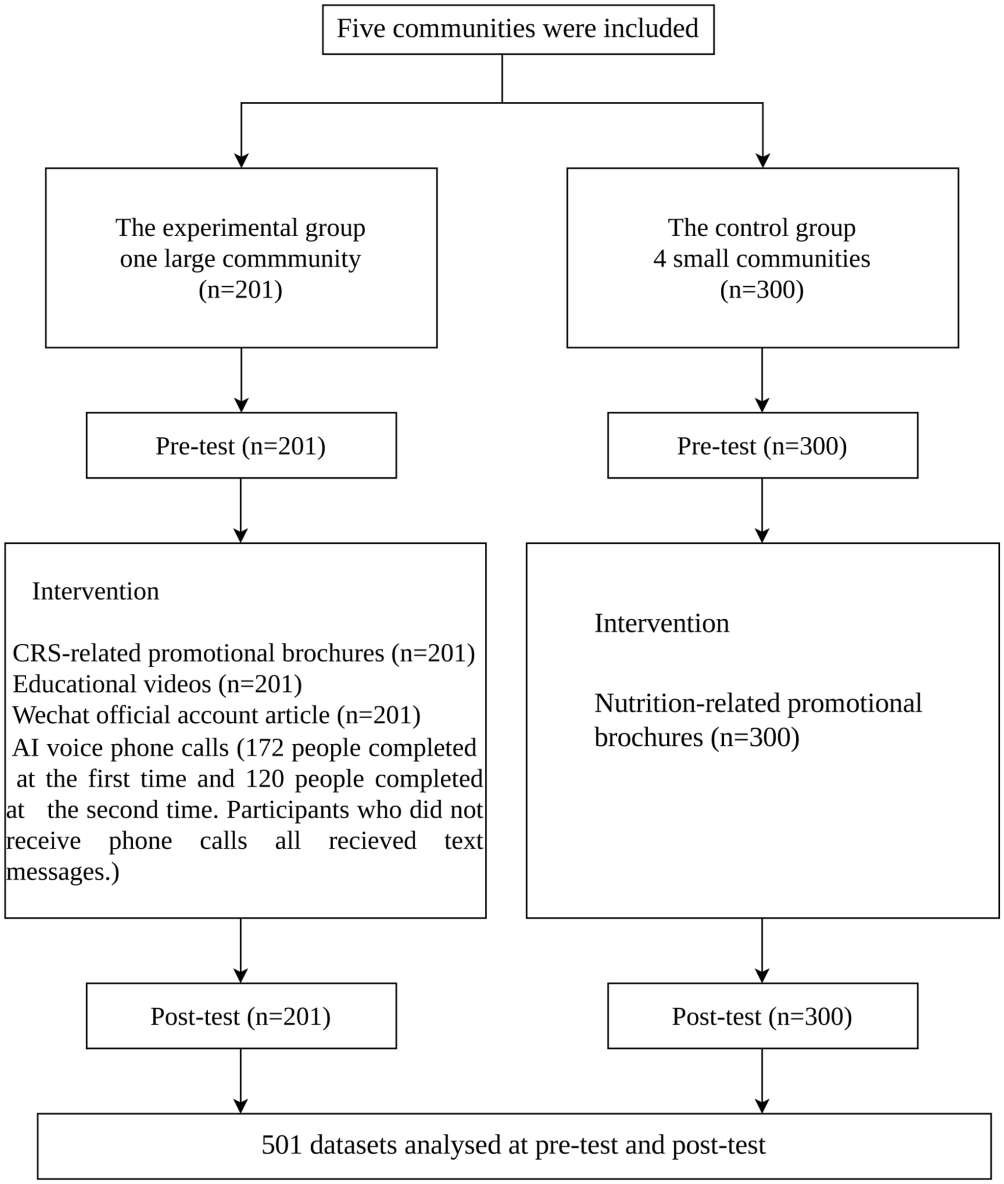
Study design flowchart.

Intervention measures lasting for six months were implemented for the intervention group: (1) September to November 2024: distribution of CRS educational brochures and health education materials once at the vaccination clinic; (2) December 2024 to January 2025: sending a health education article related to CRS to the participants once through the official WeChat public account. (3) In February and March 2025, twice AI-assisted voice calls were conducted to popularize knowledge and skills related to CRS among parents. After the calls, follow-up WeChat educational messages were sent to reinforce key CRS-related information. Participants in the control group only received educational pamphlets about dietary nutrition. Figure 1 displays the study flowchart.

The intervention consisted of a fixed-script automated voice follow-up call. The call script was developed based on findings from the pilot survey and refined by CPST technicians. Each participant in the intervention group received the same pre-recorded call, and there was no question branching. The voice interaction system verified participant identity, delivered brief CRS-related health education messages, and collected follow-up information.

The call content included:

1. identity verification and explanation of the call purpose;
2. key CRS-related safety and regulatory information;
3. follow-up on recent travel and CRS use;
4. assessment of service needs, such as installation guidance and consultation channels;
5. a brief reminder that short or infrequent trips are not valid reasons for not using a child restraint system.

To ensure intervention fidelity and replicability, the calls followed a fixed script without question branching. The complete call script has been provided in Supplementary Appendix A.

For participants who did not answer the calls, we sent text messages about correct use of CRS to ensure they received the intervention content.

### 2.5 Quality control

Quality control was implemented by staff from the district Center for Disease Control and Prevention and the community health service centers. Before the study began, all research staff received standardized training on data collection and intervention delivery. The questionnaires and intervention materials were prepared in advance and reviewed before use. During the study period, the study staff checked questionnaire completeness and monitored intervention delivery. Participants who did not answer the AI voice calls received text messages with information on correct CRS use. After the study, we cleaned the database, verified missing data items and abnormal values, and summarized and inspected intervention registration records to ensure the completeness and consistency of data and intervention processes.

### 2.6 Study outcomes

The main outcome measures were the rate of CRS ownership and the rate of consistent CRS use. CRS ownership was defined as a household possessing or providing a child restraint system for the child. The rate of consistent CRS use refers to the condition that during an average of 10 car trips, children use CRS at least 9 times.

CRS error definitions followed the Child Passenger Safety Technical (CPST) classification framework (20). We assessed two caregiver-reported CRS error indicators: selection error and orientation-related installation error. These two indicators were based on caregiver’s report of the CRS type/category and the CRS installation orientation relative to the vehicle’s direction of travel.

Selection error was determined according to the child’s age and the reported CRS type, based on NHTSA age-based recommendations (21). Among children under 1 year of age, use of a forward-facing car seat, booster seat, or booster cushion was classified as a selection error. Among children aged 1–3 years, use of a booster seat or booster cushion was classified as a selection error. For children aged 1–3 years, use of a rear-facing or forward-facing car seat may be appropriate depending on the manufacturer’s instructions; therefore, in this study, neither was classified as a selection error.

Orientation-related installation error was defined as a mismatch between the reported CRS type and its installation orientation. Rear-facing car seat were expected to be installed opposite to the direction of travel. Forward-facing car seats, booster seats, and booster cushions were expected to be installed in the same direction as travel. The corresponding questionnaire items and classification rules are provided in Supplementary Appendix B.

### 2.7 Statistical analysis

Statistical analyses were performed using R version 4.5.2. Descriptive statistics were used to summarize demographic characteristics. The Pearson’s chi-square test was used to compare the rates of CRS ownership and consistent use between the intervention and control groups.

To evaluate the effectiveness of the intervention, binary logistic regression models were constructed. This model examined the impact of the intervention on two primary binary outcomes: (1) CRS ownership (coded as 1 = owned, 0 = not owned); and (2) consistent CRS use (coded as 1 = consistent use, 0 = inconsistent use or non-use).

$$\text{logit}[\mathrm{Pr}(Y_{i}=1)]=\beta_{0}+\beta_{1}I+\beta_{2}G+\beta_{3}I\times G+\beta_{K}x_{\mathrm{ki}}$$

$Y_{i}$ represents the outcome variable (either CRS ownership or consistent use).

I represents the intervention period, coded as 0 for pre-intervention and 1 for post-intervention.

G represents the group indicator, coded as 0 for the control group and 1 for the intervention group.

I×G is the interaction term between the intervention period and group.

$x_{\mathrm{ki}}$ represents a series of covariates, including child-related factors (child age), parental factors (parental education level, household registration), vehicle-related factors (vehicle price), and travel-related factors (travel frequency, travel distance).

$\beta_{3}$ is the primary parameter of interest. It quantifies the intervention effect, representing the differential change in the outcome for the intervention group relative to the control group from the pre-intervention to the post-intervention period.

The odds ratios (ORs) and their corresponding 95% confidence intervals (CIs) were reported to quantify the strength of associations, and a *p* < 0.05 was considered statistically significant.

## 3 Results

### 3.1 Sample characteristics and distribution

A total of 501 participants were included in the study, comprising 201 in the intervention group and 300 in the control group. All participants completed the study, and no participants were lost to follow-up. The sample consisted of 264 parents of boys and 237 parents of girls. Regarding the age distribution of the children, 228 were in the 0–1 year group, 159 in the 1–2 year group, and 114 in the 2–3 year group. Additional baseline characteristics, including household registration, parental education level, vehicle price, annual family income, travel frequency, and travel distance, are presented in Table 1.

**Table 1:** Participant characteristics in the intervention and control groups.

Characteristic	Control, *n* (%)	Intervention, *n* (%)	Total, *n* (%)
Gender			
Boy	155 (51.67%)	109 (54.23%)	264 (52.69%)
Girl	145 (48.33%)	92 (45.77%)	237 (47.31%)
Child age group (years)			
0–1	126 (42.00%)	102 (50.75%)	228 (45.51%)
1–2	87 (29.00%)	72 (35.82%)	159 (31.74%)
2–3	87 (29.00%)	27 (13.43%)	114 (22.75%)
Household registration			
Shanghai	261 (87.00%)	149 (74.13%)	410 (81.84%)
Other provinces	39 (13.00%)	52 (25.87%)	91 (18.16%)
Parental education level			
Below a bachelor’s degree	14 (4.67%)	11 (5.47%)	25 (4.99%)
Bachelor’s degree	243 (81.00%)	142 (70.65%)	385 (76.85%)
Postgraduate degree or above	43 (14.33%)	48 (23.88%)	91 (18.16%)
Vehicle price (CNY)			
≤100,000	3 (1.00%)	3 (1.49%)	6 (1.20%)
>100,000 and ≤170,000	40 (13.33%)	44 (21.89%)	84 (16.77%)
>170,000 and ≤250,000	111 (37.00%)	66 (32.84%)	177 (35.33%)
>250,000 and ≤350,000	74 (24.67%)	40 (19.90%)	114 (22.75%)
>350,000	66 (22.00%)	45 (22.39%)	111 (22.16%)
Unknown	6 (2.00%)	3 (1.49%)	9 (1.80%)
Annual family Income (CNY)			
≤120,000	16 (5.33%)	13 (6.47%)	29 (5.79%)
>120,000 and ≤240,000	103 (34.33%)	67 (33.33%)	170 (33.93%)
>240,000	181 (60.33%)	121 (60.20%)	302 (60.28%)
Travel Frequency			
Once a month or less	52 (17.33%)	28 (13.93%)	80 (15.97%)
2–4 times/month	80 (26.67%)	46 (22.89%)	126 (25.15%)
2–3 times/week	138 (46.00%)	106 (52.74%)	244 (48.70%)
Daily	30 (10.00%)	21 (10.45%)	51 (10.18%)
Travel distance			
< 3 km	33 (11.00%)	53 (26.37%)	86 (17.17%)
3–5 km	66 (22.00%)	45 (22.39%)	111 (22.15%)
5–10 km	137 (45.67%)	45 (22.39%)	182 (36.33%)
≥10 km	64 (21.33%)	58 (28.86%)	122 (24.35%)
Total	300 (100.00%)	201 (100.00%)	501 (100.00%)

Data are presented as *n* (%).

### 3.2 Main intervention effects

Before the intervention, there were no significant differences between the control and intervention group in the rate of CRS ownership (87.67% versus 86.07%, *p* = 0.70) or consistent CRS use (35.67% versus 29.85%, *p* = 0.21). However, the intervention group had a higher selection error rate than the control group (81.40% versus 55.47%, *p* < 0.01), and a higher orientation-related installation error rate (56.40% versus 19.51%, *p* < 0.01) (Table 2).

**Table 2:** Differences in CRS outcomes between control and intervention groups pre-intervention.

Outcome	Control, *n* (%)	Intervention, *n* (%)	*χ* ^2^	*P*-value
Ownership				
Yes	263 (87.67%)	173 (86.07%)	0.15	0.70
No	37 (12.33%)	28 (13.93%)		
Consistent CRS use				
Yes	107 (35.67%)	60 (29.85%)	1.58	0.21
No	193 (64.33%)	141 (70.15%)		
Selection error				
No Error	110 (44.53%)	32 (18.60%)	30.42	<0.01^**^
Error	137 (55.47%)	140 (81.40%)		
Orientation-related installation error				
No Error	198 (80.49%)	75 (43.60%)	59.16	<0.01^**^
Error	48 (19.51%)	97 (56.40%)		

Data are presented as *n* (%). **p < 0.01. CRS: Child Restraint System. Percentages for CRS ownership and consistent CRS use were calculated using the total group sample as the denominator. Selection error percentages were calculated among participants with valid responses on CRS type/category. Orientation-related installation error percentages were calculated among participants with valid responses on CRS type/category and CRS installation orientation relative to the vehicle’s direction of travel.

After a 6-month intervention, the CRS ownership rate in the intervention group was 90.05%, while the rate in the control group was 91.33%. The difference was not statistically significant (*χ*^2^ = 0.11, *p* = 0.74). The rate of consistent CRS use in the intervention group was 50.75%, compared to 38.33% in the control group, with a statistically significant difference (*χ*^2^ = 7.06, *p* < 0.01). Among families who did not own a CRS, 17 out of 20 (85.00%) in the intervention group planned to purchase a CRS within the next 6 months, while only 9 out of 26 (35.00%) in the control group planned to purchase one in the future. The intention to purchase was significantly higher in the intervention group than in the control group (*χ*^2^ = 9.72, *p* < 0.01). The selection error rate was 46.67% in the intervention group and 55.35% in the control group, with no significant difference (*χ*^2^ = 3.27, *p* = 0.07). The intervention group had no orientation-related installation errors, while the control group had a rate of 12.55%, with a statistically significant difference (*χ*^2^ = 21.64, *p* < 0.01) (see Table 3).

**Table 3:** Differences in CRS outcomes between control and intervention groups post-intervention.

Outcome	Control, *n* (%)	Intervention, *n* (%)	*χ* ^2^	*P*-value
Ownership				
Yes	274 (91.33%)	181 (90.05%)	0.11	0.74
No	26 (8.67%)	20 (9.95%)		
Consistent CRS use				
Yes	115 (38.33%)	102 (50.75%)	7.06	<0.01**
No	185 (61.67%)	99 (49.25%)		
Selection error				
No Error	121 (44.65%)	96 (53.33%)	3.27	0.07
Error	150 (55.35%)	84 (46.67%)		
Orientation-related installation error				
No Error	237 (87.45%)	172 (100.00%)	21.64	<0.01**
Error	34 (12.55%)	0 (0.00%)		

Data are presented as *n* (%). **p < 0.01. CRS: Child Restraint System. Percentages for CRS ownership and consistent CRS use were calculated using the total group sample as the denominator. Selection error percentages were calculated among participants with valid responses on CRS type/category. Orientation-related installation error percentages were calculated among participants with valid responses on CRS type/category and CRS installation orientation relative to the vehicle’s direction of travel.

Logistic regression with interaction terms indicated that after intervention, the net effect on CRS ownership rate in the intervention group showed no significant statistical difference compared to the control group (OR = 0.97, 95% CI: 0.41–2.30). The ownership rate was influenced by child age, parental education level, vehicle price, and travel frequency. The ownership rate was higher for families with children aged 2–3 years compared to those with children aged 0–1 years (OR = 2.64, 95% CI: 1.41–5.24). Families with higher parental education levels showed higher ownership rates, with the highest rate observed in families with postgraduate’s degree or above (OR = 7.00, 95% CI: 2.48–20.70), compared to families with below a bachelor’s degree. Vehicle price also affected ownership rates. Families owning cars valued at 170,000–250,000 CNY showed the highest ownership rate (OR = 10.70, 95% CI: 2.03–45.80), compared to families owning vehicles priced at or below 100,000 CNY. Families with more frequent travel had higher ownership rates, with the highest rate observed in families traveling nearly every day (OR = 6.31, 95% CI: 2.18–23.30), compared to those traveling once a month or less (see Table 4).

**Table 4:** Logistic regression results for factors influencing CRS ownership rates.

Characteristics	OR value	95% CI		z-value	*P*-value
Intervention					
Pre-intervention (ref)	–	–	–	–	–
Post-intervention	1.57	0.89	2.81	1.54	0.12
Group					
Control (ref)	–	–	–	–	–
Intervention	0.91	0.51	1.65	−0.31	0.76
Intervention * Group Interaction	0.97	0.41	2.30	−0.07	0.95
Household Registration					
Shanghai (ref)	–	–	–	–	–
Other provinces	0.65	0.38	1.13	−1.56	0.12
Child age Group (years)					
0–1 (ref)	–	–	–	–	–
1–2	1.51	0.93	2.49	1.66	0.10
2–3	2.64	1.41	5.24	2.93	<0.01^**^
Parental education level					
Below a bachelor’s degree (ref)	–	–	–	–	–
Bachelor’s degree	2.68	1.23	5.69	2.54	0.01^*^
Postgraduate degree or above	7.00	2.48	20.70	3.62	<0.01^**^
Vehicle Price (CNY)					
≤100,000 (ref)	–	–	–	–	–
>100,000 and ≤170,000	4.35	0.84	18.30	1.92	0.05
>170,000 and ≤250,000	10.70	2.03	45.80	3.07	<0.01^**^
>250,000 and ≤350,000	10.50	1.94	47.80	2.96	<0.01^**^
>350,000	4.38	0.84	18.90	1.92	0.06
Unknown	1.52	0.22	9.58	0.44	0.66
Annual family Income (CNY)					
≤120,000 (ref)	–	–	–	–	–
>120,000 and ≤240,000	0.39	0.14	1.00	−1.94	0.05
>240,000	0.67	0.25	1.62	−0.85	0.40
Travel frequency					
Once a month or less (ref)	–	–	–	–	–
2–4 times/month	1.58	0.86	2.87	1.49	0.14
2–3 times/week	2.53	1.41	4.54	3.12	<0.01^**^
Daily	6.31	2.18	23.30	3.11	<0.01^**^
Travel distance					
< 3 km (ref)	–	–	–	–	–
3–5 km	0.67	0.31	1.38	−1.08	0.28
5–10 km	0.81	0.39	1.61	−0.58	0.56
≥10 km	0.60	0.29	1.21	−1.39	0.17

OR, Odds Ratio; CI, Confidence Interval; Ref, Reference group; CNY, Chinese Yuan. *p < 0.05, **p < 0.01.

After intervention, the net effect on consistent use rate in the intervention group was 2.18 times higher than in the control group, with a statistically significant difference (95% CI: 1.25–3.82). The main factors influencing the consistent use rate were household registration, travel frequency, and travel distance. Families from other provinces had lower consistent use rate than local households, with a significant difference (OR = 0.37, 95% CI: 0.23–0.56). Families with higher travel frequency had higher consistent use rate. Families traveling daily had the highest consistent use rate compared to those traveling once a month or less (OR = 3.71, 95% CI: 2.02–6.93). Families traveling longer distances (over 5 km) had a significantly higher consistent use rate compared to those traveling less than 3 km, with the highest use rate observed in families traveling 5–10 km (OR = 1.81, 95% CI: 1.16–2.85) (see Table 5).

**Table 5:** Logistic regression results for factors influencing the rates of consistent CRS use.

Characteristics	OR value	95%CI		z-value	*P*-value
Intervention					
Pre-intervention (ref)	–	–	–	–	–
Post-intervention	1.22	0.85	1.74	1.09	0.28
Group					
Control (ref)	–	–	–	–	–
Intervention	0.94	0.62	1.43	−0.28	0.78
Intervention * Group Interaction	2.18	1.25	3.82	2.74	0.01^*^
Household Registration					
Shanghai (ref)	–	–	–	–	–
Other provinces	0.37	0.23	0.56	−4.48	<0.01^**^
Child age Group (years)					
0–1 (ref)	–	–	–	–	–
1–2	0.85	0.61	1.17	−1.01	0.32
2–3	1.14	0.79	1.62	0.70	0.49
Parental education level					
Below a bachelor’s degree (ref)	–	–	–	–	–
Bachelor’s degree	0.81	0.39	1.74	−0.55	0.58
Postgraduate degree or above	0.65	0.29	1.49	−1.04	0.30
Vehicle Price (CNY)					
≤100,000 (ref)	–	–	–	–	–
>100,000 and ≤170,000	3.17	0.82	16.00	1.57	0.12
>170,000 and ≤250,000	2.82	0.74	14.20	1.41	0.16
>250,000 and ≤350,000	2.81	0.73	14.20	1.40	0.16
>350,000	1.86	0.48	9.47	0.84	0.40
Unknown	2.74	0.48	18.00	1.11	0.27
Annual family Income (CNY)					
≤120,000 (ref)	–	–	–	–	–
>120,000 and ≤240,000	0.72	0.37	1.43	−0.96	0.34
>240,000	1.32	0.69	2.58	0.83	0.41
Travel Frequency					
Once a month or less (ref)	–	–	–	–	–
2–4 times/month	0.97	0.59	1.61	−0.12	0.91
2–3 times/week	2.05	1.30	3.28	3.06	< 0.001^***^
Daily	3.71	2.02	6.93	4.17	0.01^*^
Travel distance					
< 3 km (ref)	–	–	–	–	–
3–5 km	1.03	0.65	1.66	0.14	0.89
5–10 km	1.81	1.16	2.85	2.61	0.01^*^
≥10 km	1.61	1.02	2.56	2.04	< 0.05^*^

OR, Odds Ratio; CI, Confidence Interval; Ref, Reference group; CNY, Chinese Yuan. *p < 0.05, **p < 0.01, ***p < 0.001.

## 4 Discussion

This study showed that an integrated intervention combining conventional health education with AI-assisted follow-up can effectively improve consistent CRS use. In contrast, there was no significant difference in CRS ownership between the intervention and control groups after the intervention, suggesting that the effect of the intervention on CRS ownership was limited over the short intervention period. Another possible reason is that the intervention mainly focused on CRS use reminders and safety education, rather than directly promoting CRS purchase. However, after the intervention, among families who did not own a CRS, 85.00% in the intervention group reported planning to purchase one within the next 6 months, compared with 35.00% in the control group. This suggested that the intervention may increase parents’ intention to purchase a CRS. In addition, the integrated intervention significantly reduced orientation-related installation errors. At baseline, there was no significant difference in CRS ownership or consistent CRS use between the two groups, but some imbalance was observed in selection error and orientation-related installation error. Because this study was community-based rather than randomized, some baseline differences between groups were possible. Therefore, the outcomes should be interpreted mainly in terms of changes over time.

The intervention effect observed in this study may be attributed to the combined effect of conventional health education and digitally supported follow-up, rather than any single intervention alone. Previous studies showed that public education campaigns alone, without other supporting measures, improved knowledge and attitudes only in the short term (4). In this study, AI-assisted voice calls and targeted WeChat messages were used after traditional education. This follow-up may help reinforce key safety messages among parents and encourage them to put that knowledge into practice. Yan et al. also found that reinforcement through the WeChat platform significantly increased CRS use (15).

Our study also found that socioeconomic factors (such as parental education level, household registration, child age and vehicle price) and travel-related behaviors (such as travel distance and travel frequency) were associated with CRS ownership and use. These findings are consistent with previous studies. Jin et al. found that many parents believed that CRS was unnecessary for short and infrequent trips (22). A study from Thailand reported that parents with higher education and income were more likely to use CRS, and that those who traveled more frequently were also more likely to use it (23). A study from the United States showed that parental education level was associated with CRS use, and advised that priority should be given to populations with lower education level (24). These findings all suggest that future interventions should pay more attention to families with fewer socioeconomic resources and lower health literacy, so that more targeted intervention strategies can be developed.

In developed countries such as the United States and Japan, the rate of CRS use is relatively high (25, 26). In contrast, the rate of CRS use in China remains low (27). Promotion of CRS use in China faces several challenges, including a lack of operational requirements in regulations, difficulties in enforcement, and insufficient coverage of supportive services such as CRS inspection stations. In terms of regulation, some regional regulations have specific operational requirements. For example, the regulation of Shanghai requires the use of CRS for children under 4 years of age in family vehicles. However, national regulations remain relatively general and do not provide clear age or height requirements. In terms of enforcement, strict CRS enforcement requires technical knowledge, but the number of qualified police officers remains limited. In addition, because CRS are usually installed in the rear seat, both roadside inspections and traffic cameras face difficulties in accurately identifying violations. CRS inspection stations are also an important measure for promoting correct use. Experience from high-income countries shows that inspection stations can effectively reduce CRS misuse (28–30). However, such stations are still not widely available in China, and their expansion is constrained by the shortage of trained personnel. Given these challenges, a community-based intervention that combines health education with AI-assisted follow-up may be an additional approach to promote the correct use of CRS.

This study has several limitations. First, it used a community-based, non-randomized design, so selection bias, baseline imbalance and residual confounding cannot be fully excluded, which may affect the internal validity of the effect estimates. In addition, because only one intervention community was included, the generalizability of the findings should be interpreted with caution. Second, selection error and orientation-related installation error were based on caregiver self-report rather than direct observation, which may have introduced information bias. Third, selection error was classified using the child’s age and reported CRS type without information on manufacturer’s recommended height and weight limits, so some misclassification may have occurred, particularly among children aged 1–3 years. This also limited further analysis of these errors. Fourth, this study evaluated an integrated intervention and did not assess the independent effect of the AI-assisted component. Future studies are needed to further examine the specific role of AI-assisted follow-up.

## 5 Conclusion

In this community-based study, an integrated intervention combining conventional health education with AI-assisted follow-up improved consistent CRS use and reduced orientation-related installation errors, but did not show a significant effect on CRS ownership. These findings suggest that multimodal interventions delivered in routine community services may help improve consistent and correct CRS use. This approach may serve as a useful complement to current CRS promotion strategies.

## Data Availability

All data in this article can be shared. Requests will be assessed by the Shanghai Municipal Center for Disease Control and Prevention. To request access please contact the corresponding author.

## References

[1] World Health Organization. Ten strategies for keeping children safe on the road. Geneva: World Health Organization (2015).

[2] He C Peng T Zhao YY Zhang YY Li Z Bai RB . Status and influencing factors of preschool child car passenger safety behaviors: an online survey among parents of kindergarten children in a district of Beijing. Chin J Public Health. (2023) 39:1485–9. doi: 10.11847/zgggws1140215

[3] World Health Organization. Global status report on road safety 2023. Geneva: World Health Organization (2023).

[4] World Health Organization. Occupant restraints: a road safety manual for decision-makers and practitioners (second edition). Geneva: World Health Organization (2022).

[5] Brown J Albanese B Ho C Elkington J Koppel S Charlton JL . Updated population-level estimates of child restraint practices among children aged 0-12 years in Australia, 10 years after introduction of age-appropriate restraint use legislation. Inj Prev. (2024) 30:100–7. doi: 10.1136/ip-2023-044994, 38050054

[6] Sartin EB Lombardi LR Mirman JH. Systematic review of child passenger safety laws and their associations with child restraint system use, injuries and deaths. Inj Prev. (2021) 27:577–81. doi: 10.1136/injuryprev-2021-044196, 34011513

[7] Standing Committee of the Shanghai Municipal People’s Congress. Shanghai Municipal Regulations on Road Traffic Management. Shanghai: Standing Committee of the Shanghai Municipal People’s Congress (2016).

[8] Zheng X Li R Yang H Yin D Yin T Wang L . The rate of child restraint system use among children aged under six years in China. Scand J Public Health. (2022) 50:1192–8. doi: 10.1177/14034948211036621, 34423709

[9] Wan L Wen D Ren YH Yang XR. Investigation on the use and cognition of child safety seats by parents of young children in a Western. Chin Prim Health Care. (2023) 37:23–6. doi: 10.3969/j.issn.1001-568X.2023.09.0007

[10] Cai W Lei L Zhou H Wang Y Peng J Jin Y . Child restraint system use and its associated factors in Shenzhen. Accid Anal Prev. (2021) 160:106321. doi: 10.1016/j.aap.2021.106321, 34339910

[11] Chen B Wang XH Qiu FQ Yu Y Gao SN He LH . Availability and use of child safety seats among children aged 0-3 years. China Prev Med J. (2025) 37:21–5. doi: 10.19485/j.cnki.issn2096-5087.2025.01.005

[12] Sun Y Liu T Chen J Huang J Wang X Lu M . Analysis of factors influencing the use of child restraint system by parents of children aged 0-6 years: an information, motivation, behavioral skills model-based cross-sectional study. BMC Pediatr. (2023) 23:2. doi: 10.1186/s12887-022-03827-9, 36593468 PMC9806879

[13] Wan L Wen D Wan X Ren YH Jia C Yang XR . Frequency of child restraint system use and parental knowledge of such systems in western China. Public Health Nurs. (2023) 40:655–61. doi: 10.1111/phn.13203, 37114457

[14] Keay L Hunter K Brown J Simpson JM Bilston LE Elliott M . Evaluation of an education, restraint distribution, and fitting program to promote correct use of age-appropriate child restraints for children aged 3 to 5 years: a cluster randomized trial. Am J Public Health. (2012) 102:e96–e102. doi: 10.2105/AJPH.2012.301030, 23078492 PMC3519299

[15] Yan S Yang J Fu J Ding K Ye W Chen X . Assessing an app-based child restraint system use intervention in China: an RCT. Am J Prev Med. (2020) 59:e141–7. doi: 10.1016/j.amepre.2020.02.003, 32334955

[16] Sun YR Liu T Ran N Chen JY Niu YS Wang X . Assessment of the effectiveness of parent-targeted interventions for the use of child restraint systems: a systematic review and meta-analysis. Transl Pediatr. (2022) 11:1939–48. doi: 10.21037/tp-22-560, 36643670 PMC9834943

[17] Lei H Gao R Yang J Li L. Parent-based intervention to improve child restraint use among kindergarteners in China. Am J Public Health. (2018) 108:1524–6. doi: 10.2105/AJPH.2018.304650, 30252521 PMC6187795

[18] Chen X Yang J Peek-Asa C Chen K Liu X Li L. Hospital-based program to increase child safety restraint use among birthing mothers in China. PLoS One. (2014) 9:e105100. doi: 10.1371/journal.pone.0105100, 25133502 PMC4136798

[19] Deng X Jin Y Duan LL Qi K Jiang Y Zhou HB . Survey on the awareness and use of child safety seat among 9484 cases in three Chinese cities. Chin J Woman Child Health Res. (2016) 27:551–5. doi: 10.3969/j.issn.1673-5293.2016.05.001

[20] Safe Kids Worldwide. (2018). Become A Tech. Available online at: https://cert.safekids.org/become-tech (accessed March 9, 2026).

[21] National Highway Traffic Safety Administration. Car Seats and Booster Seats. Washington, DC: National Highway Traffic Safety Administration (2026).

[22] Jin Y Deng X Ye PP Duan LL. Analysis on influence of the self-confidence, motivation and authoritative advice factors on the use of child restraint. Chin J Epidemiol. (2019) 40:1376–80. doi: 10.3760/cma.j.issn.0254-6450.2019.11.007, 31838807

[23] Vuttipittayamongkol P Muenpa R Wannapaschaiyong P. Use of child restraint systems in Thailand and factors associated with it: a cross-sectional study. Siriraj Med J. (2024) 76:11. doi: 10.33192/smj.v76i11.269751

[24] Talbot M Miller L Hafoka S. Child safety seat checks in salt Lake County: protective and risk factors. Inj Prev. (2025) 31:262–3. doi: 10.1136/ip-2023-045218 38789250

[25] Sartin EB Metzger KB Curry AE O'Malley L Pfeiffer MR Mansfield JA. Sociodemographic disparities in child restraint selection and variation in child passenger safety information sources. Accid Anal Prev. (2023) 188:107094. doi: 10.1016/j.aap.2023.107094, 37156072

[26] Morikawa M Yamada T Kogo H Sugawara M Nishikawa A Fukushi Y . Infant car seat use in Japan after the antepartum distribution of an educational leaflet: a prospective, nonrandomized controlled trial with a questionnaire survey. Traffic Inj Prev. (2020) 21:169–74. doi: 10.1080/15389588.2020.1733540, 32154734

[27] Yu Y Deng X Jin Y Duan L Peng J. Child safety seat use among 0 – 3 year old children in urban Shanghai: a cross-sectional study. Chin J Public Health. (2021) 37:668–73. doi: 10.11847/zgggws1129258

[28] Burdett BRD Starkey NJ Charlton SG. The close to home effect in road crashes. Saf Sci. (2017) 98:1–8. doi: 10.1016/j.ssci.2017.04.009

[29] Brown J Finch CF Hatfield J Bilston LE. Child restraint fitting stations reduce incorrect restraint use among child occupants. Accid Anal Prev. (2011) 43:1128–33. doi: 10.1016/j.aap.2010.12.021, 21376910

[30] Tessier K. Effectiveness of hands-on education for correct child restraint use by parents. Accid Anal Prev. (2010) 42:1041–7. doi: 10.1016/j.aap.2009.12.011, 20441811 PMC2865471

