# Supplementary material for: Effect of an artificial intelligence-assisted community-based intervention on child restraint system practices in Shanghai, China
Source: Front Public Health. 2026 Apr 20;14:1799063. doi: 10.3389/fpubh.2026.1799063 (PMC13135966; doi:10.3389/fpubh.2026.1799063)
Supplement: Supplementary file 1 [file Data_Sheet_1.pdf]

Supplementary Material

Supplementary Appendix

Contents

Appendix A: The script of AI voice call 2

Appendix B : Classiffication rules of ‘selection error’ and ‘orientation-related installation error’...................
....3

Appendix A: The script of AI voice call

The content of AI voice call intervention is as follows:

Dear parents, hello. This is the Intelligent Health Management Assistant from the Community Health Service Center. May I confirm that I am speaking to [name] ? Thank you for participating in the Shanghai Child Restraint Systems (CRS) Intervention Program. I am calling today for a follow-up survey and would like to ask you a few questions. There are no right or wrong answers—please respond based on your actual situation.

1. First question: The use of Child Restraint Systems (CRS) has been incorporated into national legislation in accordance with the latest legal provisions. Article 18 of the *Law on the Protection of Minors* clearly stipulates that parents or other guardians of minors shall take protective measures (including the proper use of CRSs) to prevent minors from sustaining traffic accident injuries. Are you aware of this regulation?
2. The newly revised *Regulations on the Protection of Minors in Shanghai*, which came into effect on March 1, 2022, explicitly requires that children under a specified age must correctly use CRSs when traveling in private passenger vehicles. Do you know about this regulation?
3. The same revised *Regulations on the Protection of Minors in Shanghai* also specify that children under a specified age are prohibited from riding in the front passenger seat of a motor vehicle. Are you aware of this rule?
4. Do you think the claim that "children do not need to use CRSs for car trips shorter than 3 kilometers" is correct?
5. How many times does your child travel by private car per week?
6. Did your child use a CRS during each of the last 10 private car trips?
7. Would you need any guidance services for the installation of a child safety seat?

Thank you for your cooperation, dear parents. When traveling with your child by car, please remember to ensure they correctly use a CRS at all times. It is a misconception to skip CRS use out of negligence, regardless of short travel distances or infrequent car trips.

Should you have any further questions, you are welcome to consult our community health service center for relevant information. Thank you for your time and answers. Wishing you good health. Goodbye.

Appendix B: Classification rules of ‘selection error’ and ‘orientation-related installation error’

Questionnaire items about the CRS selection

1. What type of CRS is your child currently using?

①Infant car seat ②Forward-facing car seat ③Booster seat ④Booster cushion ⑤Not sure

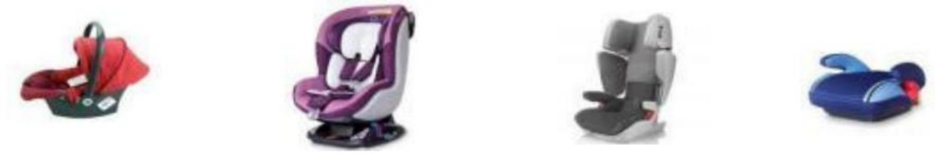

2. What is the installation orientation of your child's current car seat?

①Rear-facing ②Forward-facing ③Not sure

Table 1 Ruling Criteria of Selection error

Age group	Rear-Facing Car Seat	Forward-Facing Car Seat	Booster Seat	Booster	Not sure
<1	No	Yes	Yes	Yes	Not included
1–3	No	No	Yes	Yes	Not included

Note

For the 1–3 age group, a conservative approach is adopted: booster seats and booster are classified as selection errors. Rear-facing car seats are not considered errors, as this age group can use either rear-facing or forward-facing child safety seats, depending on the manufacturer's instructions.

Table 2 Ruling Criteria of Orientation-related installation error

Types of CRS	Same direction	Different direction	Not sure
Rear-Facing Car Seat	Yes	No	Not included
Forward-Facing Car Seat	No	Yes	Not included
Booster Seat	No	Yes	Not included
Booster	No	Yes	Not included
Not sure	Not included	Not included	Not included
